# Characterization of expressed sequence tags obtained by SSH during somatic embryogenesis in *Cichorium intybus *L

**DOI:** 10.1186/1471-2229-7-27

**Published:** 2007-06-06

**Authors:** Sylvain Legrand, Theo Hendriks, Jean-Louis Hilbert, Marie-Christine Quillet

**Affiliations:** 1 UMR USTL, INRA 1281 Stress Abiotiques et Différenciation des Végétaux Cultivés, Université de Sciences et Technologies de LILLE, Bâtiment SN2, 59655 Villeneuve d'Ascq, France

## Abstract

**Background:**

Somatic embryogenesis (SE) is an asexual propagation pathway requiring a somatic-to-embryonic transition of differentiated somatic cells toward embryogenic cells capable of producing embryos in a process resembling zygotic embryogenesis. In chicory, genetic variability with respect to the formation of somatic embryos was detected between plants from a population of *Cichorium intybus *L. landrace Koospol. Though all plants from this population were self incompatible, we managed by repeated selfing to obtain a few seeds from one highly embryogenic (E) plant, K59. Among the plants grown from these seeds, one plant, C15, was found to be non-embryogenic (NE) under our SE-inducing conditions. Being closely related, we decided to exploit the difference in SE capacity between K59 and its descendant C15 to study gene expression during the early stages of SE in chicory.

**Results:**

Cytological analysis indicated that in K59 leaf explants the first cell divisions leading to SE were observed at day 4 of culture. In contrast, in C15 explants no cell divisions were observed and SE development seemed arrested before cell reactivation. Using mRNAs isolated from leaf explants from both genotypes after 4 days of culture under SE-inducing conditions, an E and a NE cDNA-library were generated by SSH. A total of 3,348 ESTs from both libraries turned out to represent a maximum of 2,077 genes. *In silico *subtraction analysis sorted only 33 genes as differentially expressed in the E or NE genotype, indicating that SSH had resulted in an effective normalisation. Real-time RT-PCR was used to verify the expression levels of 48 genes represented by ESTs from either library. The results showed preferential expression of genes related to protein synthesis and cell division in the E genotype, and related to defence in the NE genotype.

**Conclusion:**

In accordance with the cytological observations, mRNA levels in explants from K59 and C15 collected at day 4 of SE culture reflected differential gene expression that presumably are related to processes accompanying early stages of direct SE. The E and NE library obtained thus represent important tools for subsequent detailed analysis of molecular mechanisms underlying this process in chicory, and its genetic control.

## Background

Cells in complex multicellular organisms acquire their structural and functional attributes by differentiation, a genetic program specific for a given environment of the cells. In many higher organisms, differentiation is a unidirectional and irreversible process. In higher plants, however, most differentiated cells are able to transdifferentiate, i.e. start a new differentiation pathway. Somatic embryogenesis (SE) may be considered as the ultimate form of this plant cell totipotency in that fully differentiated somatic cells are induced to regenerate new plants via a developmental pathway that resembles zygotic embryogenesis [1]. Despite considerable efforts, the processes underlying the transition from a somatic to embryogenic cell, i.e. induction, dedifferentiation, and redifferentiation, are still poorly understood [2].

For twenty years, processes related to the induction of SE in chicory are being studied in our laboratory at the cytological, physiological, and molecular level using the interspecific hybrid '474' (*Cichorium intybus *L. × *C. endivia *L.). In contrast to many agronomical important varieties of chicory, somatic embryos are readily and rapidly formed in large numbers in different explants from the hybrid '474' when cultured under constant agitation in the dark at 35°C in a Murashige and Skoog culture medium containing low concentrations of auxin and cytokinin [3]. SE is direct under these conditions, i.e. without the development of a callus, and embryos are formed from single cells [4].

Using the chicory hybrid '474' SE model system, different methods have been applied to clone genes that might be involved in the early phases of SE, or that at least could serve as markers of SE induction. The genes corresponding to the cloned cDNAs were differentially expressed in explants of the hybrid '474' during SE and not or only weakly expressed in explants of non-embryogenic *C. intybus *L. varieties cultured under the same conditions, suggesting that the expression of these genes was related to SE and not to the stress due to the culture conditions [5-8]. However, determination of causal relationships between the differentially expressed genes and SE is hampered due to the interspecific status of hybrid '474', and its complete sterility.

More recently, genetic variability with respect to the formation of somatic embryos was found present in a Hungarian landrace of *C. intybus *L., called 'Koospol', from which also the *C. intybus *L. parent of the hybrid '474' originated (M-C. Quillet, B. Delbreil, and B. Deprez, unpublished results). Upon screening plants from this landrace, embryogenic and non-embryogenic genotypes were identified, offering the possibility to introduce genetics as a tool to study the molecular mechanisms underlying the induction of SE in chicory. The plant K59 was selected as a highly embryogenic (E) genotype, and a few seeds were obtained after repeated selfing of this normally self-incompatible, and highly heterozygous, genotype. Amongst the plants grown from these seeds, the plant C15 was found to represent a non-embryogenic (NE) genotype, incapable of forming somatic embryos under our SE-inducing conditions. Sharing a similar genetic background, the genotypes K59 and its descendant C15 thus seemed an obvious choice as starting material for detailed analysis of differential gene expression during the early stages of SE in chicory.

In this study, we report the generation of an embryogenic (E) and a non-embryogenic (NE) cDNA library by applying suppression subtractive hybridization (SSH) [9] using mRNAs isolated from leaf explants of genotypes K59 and C15, cultured for 4 days under SE-inducing conditions. From the libraries 3,500 cDNA-clones were selected, sequenced, and subjected to database searches to annotate the putative functions of the representing genes. Differentially expressed genes were identified by *in silico *subtraction analysis and real-time RT-PCR. Several genes preferentially expressed in K59 seem to encode proteins involved in protein synthesis and cell division, whereas proteins encoded by genes preferentially expressed in C15 may be involved in defence. The results are discussed with respect to the quality of the libraries, and their use for future research on differential gene expression during SE in chicory.

## Results

### Cellular events during the induction of somatic embryogenesis in K59 and C15

**Figure 1:**
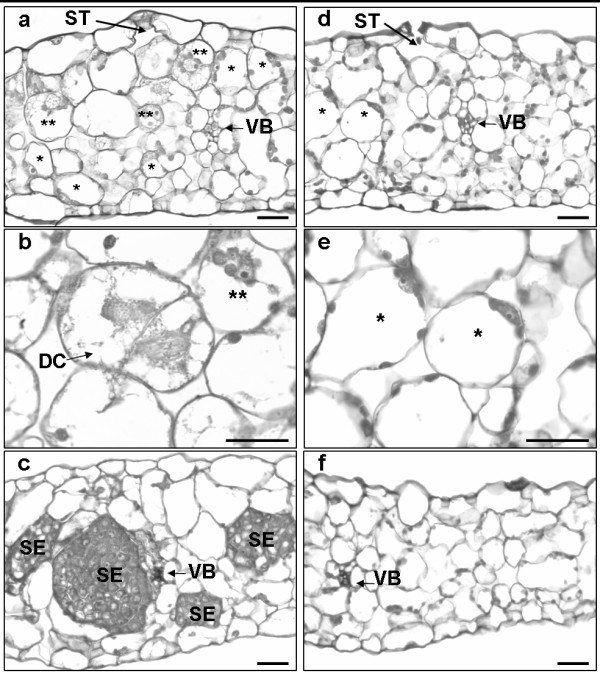
**Cytological differences in leaf explants of chicory genotypes K59 and C15 cultured under SE-inducing conditions**. Light microscopic images of stages in direct SE in leaf explants from the genotypes K59 (a, b, c) and C15 (d, e, f); semi-thin (3 μm) sections from leaf explants at day 4 (a, b, d, e) and day 8 (c, f) of culture under SE-inducing conditions stained with toluidine blue. At day 4, reactivating (*) and reactivated (**) cells can be observed in K59 (a), a well as some recently divided cells (b; DC), whereas in C15 only some reactivating cells are present (c, d). At day 8, numerous somatic embryos (SE) can be observed in K59 explants. ST = stomate; VB = vascular bundle. Bars: 30 μm.

When leaf explants from the genotypes K59 and C15 were cultivated under SE-inducing conditions as developed for the hybrid '474' [3], somatic embryos were formed in the explants from K59, but not in those from C15 (Fig. 1). Following the cellular events in explants from both genotypes during SE culture by microscopic examination of semi-thin sections, revealed that SE development in K59 was similar as described previously for the hybrid '474' [4]. The first visible response in the explants, starting after one day of culture, was cell reactivation; reactivating cells being characterized by enlarged nuclei and clearly distinguishable nucleoli. At this stage the nucleus is still oppressed against the cell wall, situated between the plasmalemma and the tonoplast of the central vacuole, and is surrounded by chloroplasts. One day later, the first reactivated cells with their nuclei positioned in the middle of the cell and surrounded by a fragmented vacuole, were observed. At day 4 of SE culture, in explants of K59 reactivating and reactivated cells were observed, as well as some first cell divisions preceding somatic embryo formation (Fig. 1a, b). In contrast, in explants from C15 only cells that seemed to have started reactivation were observed (Fig. 1d, e), albeit in lower numbers as compared to K59. Observations at day 8 of the culture showed the presence of many proembryos in the explants of K59 (Fig. 1c), whereas in the explants from C15 there was still no development of reactivated or dividing cells (Fig. 1f). From these results it was concluded that differences in mRNA levels in explants from K59 and C15 collected at day 4 of SE culture are likely to reflect differential gene expression related to processes accompanying the early stages of SE in K59.

### Generation of SSH from an embryogenic and a non-embryogenic genotype

Messenger RNAs isolated from leaf explants of K59 and C15, collected at day 4 of culture under SE inducing conditions, were used to construct two subtractive cDNA-libraries by applying SSH. The E library, a library supposed to be enriched in cDNAs representing genes preferentially expressed during SE, was obtained by using cDNAs from K59 mRNAs as 'tester' and cDNAs from C15 mRNAs as 'driver'. The NE library was obtained by reversing 'tester' and 'driver' mRNA.

Sequencing was carried out for about 3,500 cDNAs clones randomly selected from both libraries: 2,000 from the E library and 1,500 from the NE library. After removing bad quality sequences, a total of 1,944 ESTs from the E library and 1,404 from the NE library were conserved for further analyses. The average length of the 3,348 ESTs was 456 bp, and the GC content was equal to 45% (Tab. 1).

**Table 1:** Summary of *Cichorium intybus *ESTs

Total ESTs	3,348
ESTs from the E library	1,944
ESTs from the NE library	1,404
Average sequence size (bp)	456
Average sequence size of annotated ESTs (bp)	471
Average sequence size of ESTs without match	354
Average GC content (%)	45
Number of original clusters (OC)	2,302
Number of contigs	189
Maximum number of genes	2,077
Genes represented only in the E library	1,061
Genes represented only in the NE library	730
Genes represented in both libraries	286

A database named E/NE db was generated from all ESTs from the E and NE library. To generate OCs (Original Clusters, regrouping identical ESTs) two successive criteria were applied. First, all 3,348 sequences from the E/NE db were submitted to a BlastN search (E-value ≤ E^-30^) against this database. The sorted sequences were grouped in 2,174 primary OCs (encoded OC0001 – OC2174). Next, sequences in primary OCs containing more than one EST were aligned and their mutual sequence identity determined. OCs containing ESTs that all shared at least 95% identity over a contiguous sequence of 150 bp retained their primary code. OCs that contained two or more groups of ESTs by this criterion were split in 2 or more OCs, respectively, each identified by the primary code followed by a letter (e.g. OC0603_a and OC0603_b; cf Tab. 3, and Additional file 1). This analysis revealed a total of 2,302 OCs, of which 1764 (52.7% of the total number of sequences) were singletons, and 538 contained between 2 and 40 ESTs.

**Table 3:** *In silico *screening and transcriptional analysis by real-time RT-PCR.

			*In silico *analyses				real-time RT-PCR		
				
Acc. nb	Contig/OC	Putative function	E	NE	P	Pred	p-value	ER 1	ER 2
DT212490	Cont0080	vegetative storage protein, VSP [C. intybus]	5	0	]0.08;0.07[	E			
DT212771	Cont4942	elongation factor EF-2 [P. sativum]	10	0	]0.006;0.005[	E	0.09435	-0.18	0.38
DT212374	Cont9008	Shaggy-like kinase tetha [A. thaliana]	5	0	]0.08;0.07[	E			
DT212650	OC0603_a	methionine synthase [S. tuberosum]	6	0	]0.05;0.04[	E			
DT212300	OC1814	No hits found	5	0	]0.08;0.07[	E	<0.000001*	2.00*	1.90*
DT212502	OC1929	G protein beta subunit [N. plumbaginifolia]	5	0	]0.08;0.07[	E	<0.000001*	1.25*	1.24*
DT213897	Cont0011	catalase 3 [H. annuus]	4	8	]0.09;0.08[	NE	<0.000001*	-3.85*	-3.88*
DT211305	Cont0015	L-asparaginase [G. max]	0	3	]0.07;0.06[	NE	0.03694	0.18	0.40
DT211671	Cont0026	ss-1,3-glucanase [C. intybus × C. endivia]	0	3	]0.07;0.06[	NE	<0.000001*	-2.87*	-4.10*
DT213305	Cont0060	3-hydroxy-3-methylglutaryl coenzyme A reductase [S. tuberosum]	8	15	]0.03;0.02[	NE	<0.000001*	-2.30*	-2.03*
DT212144	Cont0082	putative argininosuccinate synthase [A. thaliana]	0	4	]0.03;0.02[	NE	0.00095*	1.23*	0.70*
DT210848	Cont0112	heat shock protein 70 like protein [A. thaliana]	0	3	]0.07;0.06[	NE			
DT211362	Cont0201	putative auxin-induced protein [A. thaliana]	0	5	]0.02;0.01[	NE	<0.000001*	-1.88*	-2.03*
DT211152	Cont0206	fasciclin-like AGP 12 [P. alba × P. tremula]	0	4	]0.03;0.02[	NE	0.00538	-0.25	-0.40
DT211657	Cont1237	pre-pro-cysteine proteinase [L. esculentum]	0	4	]0.03;0.02[	NE	<0.000001*	-2.32*	-3.00*
DT211016	Cont1242	unknown protein [A. thaliana]	0	3	]0.07;0.06[	NE			
DT210870	Cont9022	unknown yeast pheromone receptor-like protein AR781 [A. thaliana]	0	3	]0.07;0.06[	NE			
DT213289	Cont9039	metallothionein 1 [A. tripolium]	14	26	]0.004;0.003[	NE	<0.000001*	-2.45*	-2.45*
DT210772	OC0003_b	glutathione S-transferase GST 13 [G. max]	0	4	]0.03;0.02[	NE			
DT212623	OC0023_a	glutathione transferase [H. muticus]	3	11	]0.007;0.006[	NE	<0.000001*	-2.78*	-2.85*
DT211055	OC0168	1-aminocyclopropane-1-carboxylate oxidase [L. esculentum]	0	9	<0.001	NE	<0.000001*	-3.87*	-3.80*
DT210897	OC0178	putative ribosomal S29 protein [A. thaliana]	0	3	]0.07;0.06[	NE	<0.000001*	1.15*	0.70*
DT210805	OC0344	unknown protein [A. thaliana]	0	3	]0.07;0.06[	NE	<0.000001*	-1.35*	-1.68*
DT211689	OC0378_b	putative 60S ribosomal protein L5 [O. sativa]	0	3	]0.07;0.06[	NE			
DT214125	OC0533b	lipid transfer protein precursor [M. domestica]	2	7	]0.04;0.03[	NE	0.06214	-0.18	-0.25
DT211562	OC0559	putative phosphatase type 2C [A. thaliana]	0	3	]0.07;0.06[	NE	0.01554	-0.25	-0.25
DT211372	OC0631	No hits found	0	3	]0.07;0.06[	NE	0.03982	0.20	0.20
DT211599	OC0686	unknown protein [A. thaliana]	0	4	]0.03;0.02[	NE	<0.000001*	-3.37*	-3.23*
DT211633	OC0696	aquaporin [H. annuus]	0	3	]0.07;0.06[	NE	<0.000001*	-3.72*	-3.52*
DT211922	OC0724	cellulose synthase family protein [A. thaliana]	0	3	]0.07;0.06[	NE	<0.000001*	-2.60*	-2.47*
DT211365	OC0727_b	G protein beta subunit-like [M. sativa]	0	5	]0.02;0.01[	NE			
DT210839	OC0758	putative RNA-binding protein [A. thaliana]	0	5	]0.02;0.01[	NE	0.01647	0.08	0.18
DT212897	OC1229	sucrose synthase isoform I [D. carota]	1	5	]0.05;0.04[	NE	0.30612	0.08	-0.32
DT212813	OC0311_a	acidic ribosomal protein P0 [G. max]	6	2			<0.000001*	1.62*	1.68*
DT212479	OC0378_c	60S ribosomal protein L5 [C. sativus]	1	3			<0.000001*	0.58*	0.85*
DT211177	OC0805_a	putative 60S ribosomal protein L35 [A. thaliana]	0	1			0.00001*	1.10*	0.73*
DT212655	OC1088	ribosomal protein L12 [C. intybus]	1	1			0.00013*	1.25*	0.95*
DT213187	OC1549_a	40S ribosomal protein S17 [L. esculentum]	2	1			<0.000001*	1.72*	1.55*
DT212250	OC2155_a	P40-like ribosomal protein [D. carota]	3	0			0.00003*	0.58*	0.82*
DT212554	Cont0001	glyceraldehyde-3-phosphate dehydrogenase [S. alba]	9	2			<0.000001*	1.20*	0.97*
DT210799	Cont0006	1-aminocyclopropane-1-carboxylate oxidase [P. hortorum]	2	5			<0.000001*	-3.82*	-4.28*
DT211255	Cont0024	seed specific protein Bn15D17A [B. napus]	3	5			0.25124	0.00	0.13
DT211067	Cont6402	putative leaf development protein Argonaute [A. thaliana]	1	1			<0.000001*	1.37*	1.37*
DT210888	OC0071	14-3-3 protein [S. tuberosum]	0	1			<0.000001*	-1.78*	-2.05*
DT210862	OC0172	embryogenic callus protein 181 [D. carota]	0	2			0.56355	-0.28	0.05
DT211413	OC0565_a	acetoacetyl-CoA thiolase [A. thaliana]	0	2			<0.000001*	-2.52*	-2.42*
DT212452	OC0603_b	cobalamine-independent methionine synthase [S. scutellarioides]	7	1			<0.000001*	-1.90*	-1.57*
DT212234	OC0687	arabinogalactan protein [D. carota]	7	1			<0.000001*	6.73*	6.82*
DT212237	OC0834	chromatin remodeling factor CHD3 [A. thaliana]	1	0			0.00042	0.50	0.50
DT212556	OC1068	leaf senescence protein-related (YLS7) [A. thaliana]	2	0			<0.000001*	-1.55*	-1.38*
DT212596	OC1155	xyloglucan endotransglycosylase [M. domestica]	2	0			0.00011	-0.57	-0.52
DT212803	OC1211	BTB/POZ domain-containing protein [A. thaliana]	2	0			0.00365	0.38	0.42
DT213079	OC1347	putative ethylene response element binding protein [N. tabacum]	1	2			0.00007*	-1.45*	-1.22*
DT212789	OC1427_b	Cell division control protein 48 homolog E [A. thaliana]	2	0			0.00001*	0.60*	0.60*
DT213276	OC1785_a	H(+)-transporting ATPase [P. vulgaris]	2	0			0.00002*	-0.87*	-0.93*
DT212233	OC1796	ring domain containing protein [C. annuum]	10	8			<0.000001*	-2.18*	-2.35*
DT212829	OC1960	tuber-specific/sucrose-responsive element binding factor [S. tuberosum]	4	0			0.00021	-0.15	-0.65

Contig/OC: contig or OC (Original Cluster regrouping identical ESTs) number. Putative functions were determined by a BlastX search (E-value ≤ E^-5^) against the non redundant GenBank database. E and NE: number of ESTs from the E and the NE library, respectively; p: probability related to the *in silico *prediction calculated according to Audic and Claverie [25]. Pred (prediction) indicates in which genotype (E or NE) the gene was found preferentially expressed. ER: expression ratios (log_2_) between the embryogenic and the non-embryogenic genotype at day 4 of SE culture estimated by real-time RT-PCR. ER1 and ER2 designate the expression ratios obtained from 2 independent first strand cDNA synthesis reactions (see Methods). The asterisk indicates genes that were found to be differentially expressed according the thresholds applied (see Methods).

### Comparison of chicory ESTs with sequences of other species allowing the formation of contigs

Direct determination of the number of genes represented by the 2,302 OCs identified was not possible since the digestion of cDNA with *Rsa*I during the SSH procedure left no overlapping sequences, and thus prevented the construction of contigs. To identify OCs that potentially represented the same gene, sequential BlastN searches (E-value ≤ E^-30^) were performed against assembled ESTs from lettuce (assembled ESTs from CGPD: Compositae Genome Project Database, [10]), sunflower (assembled ESTs from CGPD), and *Zinnia elegans *L. (assembled ESTs from PGDB: Plant Genome Database, [11]) (Tab. 2). The hierarchy of the searches was determined by the botanical proximity of the species; chicory and lettuce belonging to the tribe Lactuaceae of the subfamily Cichorioideae of the Asteraceae, whereas sunflower and *Z. elegans *belong to the subfamily Asteroideae [12]. In parallel, BlastX searches were performed using the non-redundant (NR) GeneBank database [13], and the Arabidopsis translated coding sequences [14].

**Table 2:** Comparison of *Cichorium intybus *ESTs with sequences from other species

Database		Number of sequences	Number of submitted chicory ESTs	Blast type (E-value)	Number of matches	% of matches
Asteraceae	Lettuce (CGPD)	19,523 assembled ESTs	3,348	N (1E-30)	2,040*	69
	Sunflower (CGPD)	18,031 assembled ESTs			202*	
	Zinnia (PGDB)	15,859 assembled ESTs			71*	
Arabidopsis (AtGDB)		26,719 translated coding sequences	3,348	X (1E-5)	2,905	87
GeneBank NR		>100 gigabases	3,348	X (1E-5)	2,794	84

*The 3348 ESTs of chicory were sequentially submitted to BlastN searches against assembled ESTs from lettuce, sunflower and zinnia.

The results of the BlastN searches in the Asteraceae databases showed a high proportion of 'no hits found', *i.e*. 1035 ESTs of chicory (31%) were not represented in Asteraceae databases. In comparison, only 13–17% of the chicory ESTs did not match to any sequence in the Arabidopsis and Genebank NR databases (Tab. 2). Furthermore, the results quite often suggested that OCs that were clearly distinguished by our OC-criteria, as well as by the results of the BlastX searches, matched to the same Asteraceae contig. Taken together, this clearly indicated that the CGPD and PGDB databases were not yet sufficiently exhaustive to represent the Asteraceae transcriptomes.

It was therefore decided to use the results of the BlastX searches to assemble the chicory OCs into contigs. In a first attempt, OCs and singletons with the same best hit (E-value ≤ E^-5^) in the AtGDB were considered to represent the same gene. However, ESTs grouped in different OC, because they had less than 95% sequence identity, were sometimes found to match with the same Arabidopsis coding sequence. In these cases very often each OC matched with a different sequence in the NR GeneBank database (see Additional file 1). This may be indicative for a higher number of duplicated genes in chicory than in Arabidopsis. These analyses led to the formation of 189 contigs by the regrouping of 111 OCs with at least 2 ESTs, and 303 singletons. Together with the remaining 1,888 OCs, we estimated that the 3,348 ESTs selected from the E and NE library represent at maximum 2,077 genes (see Additional file 1). From these 2,077 annotated genes, 1,061 genes (51%) were composed of ESTs exclusively originating from the E library, 730 genes (35%) of ESTs exclusively originating from the NE library, and 286 genes (14%) of ESTs present in both libraries.

The total number of genes is probably overestimated, since part of the OCs and contigs containing ESTs without significant matches may correspond to genes already accounted for, because they represent untranslated mRNA regions. If we consider OCs composed of non-matching ESTs to be part of genes already accounted for, and when we omit the criterion of 95% identity over a contiguous sequence of 150 bp to divine an OC, a minimum of 1,698 genes was obtained.

### Annotation and functional classification of ESTs

Annotation of the ESTs using BlastX searches against sequences in NR GeneBank database, revealed that 55% of the ESTs had a high similarity with the best match (E-value ≤ E^-30^), and 32% a moderate similarity (E^-30 ^to E^-5^). The 13% remaining with an E-value ≥ E^-5^, or with no match found, was classified as 'no hits found'. The lack of sequence homology may be related to the average length of 354 bp for these ESTs, about 120 bp less than the average sequence length of 471 bp for annotated ESTs (Tab. 3) [15].

**Figure 2:**
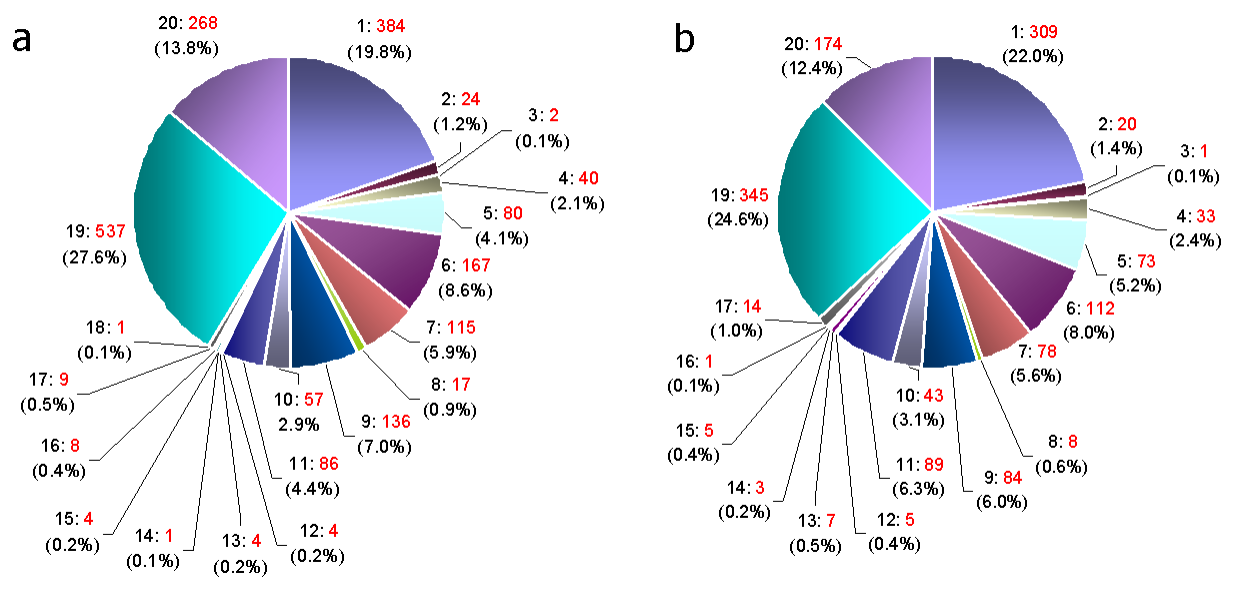
**Distribution of the putative functions of 3348 ESTs from the E (a) and NE (b) library**. Classification was performed according to the Munich Information centre for Proteins Sequences (MIPS) functional catalogue using BlastX search results against Arabidopsis coding sequences from the Arabidopsis genomic database (AtGDB). 1: Metabolism; 2: Energy; 3: Storage protein; 4: Cell cycle and DNA processing; 5: Transcription; 6: Protein synthesis; 7: Protein fate (folding, modification, destination); 8: Protein with binding function or cofactor requirement (structural or catalytic); 9: Cellular transport, transport facilitation and transport routes; 10: Cellular communication/signal transduction mechanism; 11: Cell rescue, defense and virulence; 12: Interaction with the cellular environment; 13: Interaction with the environment (Systemic); 14: Transposable elements, viral and plasmid proteins; 15: Cell fate; 16: Development (Systemic); 17: Biogenesis of cellular components; 18: Cell type differentiation; 19: Function unknown (regrouping Subcellular localization, Classification not yet clear cut, and Unclassified proteins); 20: No hits found.

The distribution of the putative functions of the annotated genes was similar for both libraries (Fig. 2), the major classes being 'function unknown' (26%) (regrouping 'subcellular localization', 'classification not yet clear cut', and 'unclassified proteins'), 'metabolism' (21%), 'protein synthesis' (8%), 'cellular transport, transport facilitation and transport routes' (7%), 'protein fate' (6%) and 'cell rescue, defence and virulence' (5%).

From the 279 ESTs representing genes related to protein synthesis, 202 (6% of the total number of ESTs) represent genes encoding ribosomal proteins: 122 and 79 ESTs from the E and the NE library, respectively. In comparison, only 1.3% of 112,500 ESTs for Arabidopsis were found to represent genes encoding ribosomal proteins [16].

The most represented genes in our libraries are a metallothionein (OC9039) with 40 ESTs (14 and 26 from the E and NE library, respectively) of the 175 ones related to 'cell rescue, defence and virulence' and a gene encoding a 3-hydroxy-3-methylglutaryl coenzyme A reductase (Cont0060) related to 'metabolism' and represented by 8 ESTs in the E library and 15 in the NE library.

The distributions of the putative functions of the annotated genes, except for those encoding ribosomal proteins, resembled those reported for genes or cDNAs in Arabidopsis and other plant species [17-19], including the about 25% sequences with undetermined functions. Apparently the SSH procedure had not effected an enrichment of ESTs representing genes implemented in particular functions in one of the genotypes, at least not at this level of functional assignment.

### *In silico *screening and real time RT-PCR

**Figure 3:**
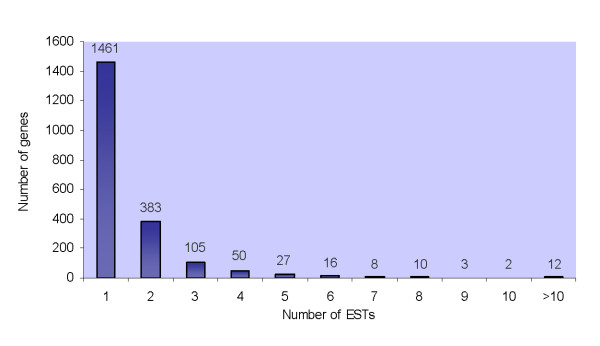
**Distribution and number of assembled sequences**.

The large proportion of the annotated genes represented by single ESTs (1,461 of 2,077, i.e. 70%) (Fig. 3) seems to indicate an efficient normalisation of the libraries; only 3% of the genes were represented by more than 5 ESTs. Furthermore, only 14% of the annotated genes in the different functional groups were represented by ESTs from both libraries (Tab. 1), as could be expected for an *ad random *selection of the ESTs from libraries that were effectively normalised.

To verify further the efficiency of the normalisation realised by the SSH procedure, we performed an *in silico *subtraction, or 'digital northern', on all the ESTs from our libraries. This analysis is based on the relation between the abundance of ESTs in a cDNA library and the differential expression of the corresponding genes [20,21], and is ordinarily performed on ESTs in cDNA libraries that are not normalized [22,23]. Inversely, applying this analysis may provide a measure for the normalisation achieved, and as normalization is nearly always imperfect, in particular for very abundant transcripts [24], it may also reveal ESTs representing genes preferentially expressed in the E or NE genotype.

Using the significance test of Audic and Claverie [25], it was found that from the 2,077 annotated genes, only 33 (1.6%) were expected to be differentially expressed; 6 genes preferentially expressed in the E genotype, and 27 in the NE genotype (Tab. 3). For 24 of the 33 predicted genes the abundance of transcripts was measured by real-time RT-PCR (Tab. 3). The results confirmed the differentially expression of 14 genes, i.e. 2 preferentially expressed in explants from the E genotype, and 12 in explants from the NE genotype. For 2 genes that were predicted to be preferentially expressed in the NE genotype, it was found that they were actually preferentially expressed in the E genotype. The remaining 8 genes were found to be not differentially expressed.

The relative low number of differentially expressed genes predicted by *in silico *subtraction suggested a high efficiency of the normalisation realised by SSH. It was reported that normalisation and enrichment by SSH is ineffective for abundant transcripts in tester or driver samples [24], leading to an elevated number of background clones. Indeed, the real-time RT-PCR experiments showed that in comparison to the level of transcripts for actin-2, transcripts of some genes were abundant (> 100-fold higher than actin-2) in explants of both K59 and C15, where as for other genes the level of transcripts was considerably lower (< 50-fold) than for actin-2 (data not shown). These results indicated that there was no relation between transcript level and EST representation in the libraries.

For another set of 24 genes, some of them selected because they have been reported to play key roles during early stages of somatic embryogenesis or during plant development (e.g. OC0687: arabinogalactan protein [26]; Cont6402: Argonaute [27]), others because they were represented by several ESTs in the libraries (e.g. Cont0001 glyceraldehyde-3-phosphate dehydrogenase; OC1796: ring domain containing protein; OC0311_a, OC0378_c: ribosomal proteins), the abundance of transcripts was also measured by real-time RT-PCR (Tab. 3). This led to the identification of an additional 18 differentially expressed genes; 10 in the E genotype, and 8 in the NE genotype (Tab. 3).

## Discussion

An E and a NE cDNA-library were generated by SSH using mRNAs isolated from leaf explants from two chicory genotypes differing in SE capacity cultured for 4 days under SE-inducing conditions, and a total of 3,348 ESTs from both libraries turned out to represent a maximum of 2,077 genes. Real time RT-PCR analyses of the expression of 48 annotated genes revealed that after 4 days of culture under SE-inducing conditions, 14 genes were preferentially expressed in the E genotype, and 20 genes in the NE genotype (Tab. 3). This indicated that the E and NE library contain ESTs representing genes differentially expressed in K59 or C15, even though ESTs found present in one library not necessarily represented a gene exclusively expressed in the corresponding genotype. In addition, some of the differences in gene expression between the two lines might be the result of genetic differences that have nothing to do with SE capacity. As we intend to use ESTs from both libraries for future studies on gene expression during SE, the most important contribution of SSH in the construction of the libraries was probably the normalisation achieved, heightening the possibility to find ESTs representing feebly expressed genes.

We chose to construct the E and NE library using mRNAs isolated from explants of K59 and C15 cultured for 4 days under SE conditions on the basis of cytological observations, in particular the occurrence of the first cell divisions in explants of K59. Seven genes encoding ribosomal proteins were tested by real-time RT-PCR, and were all found to be preferentially expressed in explants of K59, the embryogenic genotype (Tab. 3). This probably reflects ribosome biogenesis required for the preparation of cells in the explants to enter the SE transdifferentiation pathway, and in particular to reinitiate cell divisions. The relation between augmented expression of genes encoding ribosomal proteins and cell divisions has been documented in several studies (e.g. [28,16]). In *Z. elegans*, many genes encoding ribosomal proteins were found to be preferentially expressed during the transdifferentiation of mesophyll cells into xylem cells [18], and in aspen relative high numbers of ESTs representing ribosomal protein genes were reported for cDNA libraries from meristematic tissues [29,30]. In addition to the ribosomal protein-encoding genes, preferential expression in K59 was detected for 2 genes implicated in cell cycling: a gene encoding a CDC48-like protein (OC1427_b) [31] and a G protein beta subunit-like protein (OC1929) [32] (Tab. 3).

The above results indicated that the preferential expression of genes implicated in cell division in K59 concurs with the cytological observations. Cells in the explants of K59 that enter the cell division cycle have lost their original identity, and most of them seem to enter the SE pathway thereafter. A gene (Cont6402) encoding a protein having a high homology with the protein Argonaute 1 (AGO1) was found to be expressed preferentially in the explants of K59 at day 4 of SE culture. In Arabidopsis, *ago1 *mutants present loss of stem cell maintenance and failure of axillary meristem formation [33], and it was shown that *AGO1*, together with *ZWILLE/PINHEAD*, regulates stem cell maintenance via *SHOOT MERISTEMLESS *(*STM*) [27]. A gene homologous to *STM *has been identified in chicory and was shown to be differentially expressed early during SE (S. Da Silva and M-C. Quillet, unpublished results). Furthermore, 2 ESTs from the E library (DT212395 and DT13465; see Additional file 1) were found to represent a gene homologous to *ZWILLE/PINHEAD *in Arabidopsis. The early expression during SE of genes regulating stem cell maintenance may indicate that they also play a role in the transdifferentiation process accompanying SE (cf. [2]).

Another interesting result may be the preferentially expression in K59 of a gene (OC0687) putatively encoding an arabinogalactan protein (AGP) similar to DcAGP1 from carrot. DcAGP1 encodes a non-classical AGP with strong similarity to a family of basic proline-rich proteins [34]. AGPs are supposed to be involved in many signaling pathways [35], and were reported to be essential for the formation of somatic embryos in chicory [26].

The cytological studies indicated that in C15 cells reacted differently to the SE-inducing culture conditions, possibly by failing to progress in cell reactivation (Fig. 1). The differences in gene expression between C15 and K59 observed at day 4 of SE culture suggests that in contrast to the opportunistic response as observed for K59, cells in the explants from C15 reacted to the stresses applied by a defensive response. This was illustrated by the preferential expression in C15 of genes involved in the ethylene signalling pathway: two genes encode ACC oxidases (Cont0006 and OC0168), and a gene encodes an ethylene response element binding protein (OC1347). Some other genes preferentially expressed in C15, also related to defence, encode a metallothionein (Cont9039), a glutathione transferase (OC0023_a), and a leaf senescence-related protein (OC1068) [36,37].

## Conclusion

The E and NE cDNA libraries described in this paper will be important new tools in our ongoing efforts to unravel the molecular mechanisms underlying the early stages of direct somatic embryogenesis in chicory. None of the genes identified in this study has been identified as such previously in our laboratory [5-8]. This is probably due to the limited number of clones from our libraries that were sequenced, to the differences in the timing and way of selection, and/or differences between the E and NE genotypes used for screening. The results of the real-time PCR analysis showed that our libraries contain ESTs representing genes differentially expressed in the E genotype K59 and the NE genotype C15. It remains to be established, however, which of these genes are implicated in the different responses of the explants from both genotypes upon SE culture conditions, and in particular in the early stages of SE. A transcriptional analysis by cDNA microarray is currently performed for explants of K59 and C15 during the first 6 days of SE culture. This should lead to the identification of genes differentially expressed during SE, and their expression patterns may provide clues on their roles in this process. Furthermore, preliminary experiments indicate that the number of SE formed in explants from plants in progenies obtained after crossing K59 with a compatible low embryogenic genotype shows a continuous quantitative distribution, i.e. behaves as a quantitative trait. These plants are polymorphic for a large number of molecular markers, and a molecular genetic map for this progeny has been realized in our laboratory. This will serve to identify quantitative trait loci (QTL) for SE, as well as to map genes differentially expressed during SE. Co-localization of genes differentially expressed during SE with QTL for this process may help to identify those genes of which the expression is causally implicated in direct SE in chicory.

This report also presents a medium scale sequencing of cDNAs representing genes in chicory. In fact, of the 3,348 ESTs selected from the E and NE library (additional file 1), only 13 showed homologies to 11 chicory sequences of the total 218 entries for chicory already present in the GeneBank NR database. Though modest in comparison to databases for some other Asteraceae, like lettuce, sunflower, and *Z. elegans*, our database for chicory may serve as a source for comparative studies in this important plant family.

## Methods

### Somatic embryogenesis culture, tissue collection and RNA extraction

The *Cichorium intybus *embryogenic (K59) and non-embryogenic (C15) genotypes were grown in the greenhouse, and maintained by vegetative propagation. Leaves from six-leaves stage plants were surface sterilized, and cut up in fine strips (2 cm × 0.2 cm). Each culture contained 15 explants from a single leaf in 20 ml M17S20 culture medium [3], and was placed in darkness at 35°C under constant agitation (80 rpm). Explants were collected at day 4 of SE culture and RNA from each culture was extracted using the Tri reagent kit (Euromedex) according to the instructions of the manufacturer. RNA integrity was checked by capillary electrophoresis (Agilent 2100 Bioanalyser, Agilent Technologies), and RNA quantities were calculated from the optical density at 260 nm.

### Microscopy

Leaf explants were fixed in a formaldehyde/acetic acid/ethanol solution (3.5/6.5/90, v/v/v), dehydrated through a range of increasing concentrations of ethanol, infiltrated with JB4 wax (Polysciences), and sectioned at 3 μm with a Leica RM 2065 microtome. After staining with a 0.5% (w/v) aqueous solution of toluidine blue, the sections were examined by light microscopy (Olympus).

### Construction of the subtractive cDNA libraries

RNA isolated from the explants of 6 and 9 independent cultures of K59 and C15, respectively, were pooled in order to create the SSH libraries. Poly(A)^+ ^RNA was extracted using the Quick Messenger RNA kit (Talent) according to the manufacturer's instructions. Two subtractive libraries were generated using the PCR Select cDNA Subtraction Kit (Clontech), according to established protocols, using 4 μg of poly(A)^+ ^RNA for the generation of first strand cDNA. The E library was obtained using the cDNA of K59 (embryogenic) and C15 (non-embryogenic) genotypes as 'tester' and 'driver', respectively. To create the NE library, 'tester' and 'driver' cDNA were reversed. PCR-amplified subtracted cDNA were cloned in pGEM-T vector (Promega), transformed into JM109 competent cells (Promega), and plated on LB plates containing ampicillin (100 μg/ml), X-gal (80 μg/ml), and IPTG (0.5 mM). Transformed white colonies were picked and grown overnight in LB containing ampicillin (50 μg/ml) in 96-well plates at 37°C and 180 rpm. Glycerol was added to obtain 30% (v/v), and the plates were stored at -80°C. Prior to sequencing, clones were transferred into LB 96 deep well plates and placed overnight at 37°C and 180 rpm. Plasmids were isolated from the overnight-grown bacterial cultures using the Plasmid Miniprep96 Kit (Millipore). Single-run sequencing was carried out in an ABI3700 DNA Sequencer (Applied Biosystems) using the universal T7 primer.

### EST analysis

ESTs were cleaned of vector and primers sequences using a Perl script (see Additional file 2, script 1). A total of 3,422 EST sequences have been submitted [GenBank: DT210770 to DT214189, DT317741 and DT317742]. Identical ESTs from the E and/or the NE library were identified using the local BlastN search included in the BioEdit sequence alignment editor [38]. To assemble ESTs into contigs, BlastN and BlastX searches were performed against EST contigs of different Asteraceae species and against sequences of the non-redundant (NR) GeneBank database and against the Arabidopsis translated coding sequences (see results). Perl scripts were used to retrieve information from the Blast output files (see Additional file 2, scripts 2 and 3). Sequences were annotated using the results of BlastX searches, and were classified according to the MIPS (Munich Information Centre for Proteins Sequences) functional catalogue [39]. Significance levels for *in silico *analysis of differential EST abundance between E and NE library were computed using the statistical program of Audic and Claverie [25,40].

### Real-time RT-PCR

One microgram of total RNA from each genotype was reverse transcribed using the First Strand Synthesis Kit (BioRad). Fourty eight gene primer pairs were designed (see Additional file 3) by using the Beacon Designer software (Biosoft). Real-time PCR were carried out with the Quantitech SYBR green kit (Qiagen) in a final volume of 20 μl, including 375 nM of each primer, and 5 μl of a 5-fold dilution of first strand cDNA. PCR reactions were performed in 96-well optical reaction plates (ABgene) using an iCycler iQ apparatus (Biorad). The reactions were heated for 10 min to 95°C followed by 50 cycles of denaturation for 30 sec at 95°C and annealing-extension for 45 sec at 60°C. For each pair of primers, the PCR efficiency (e) was calculated using different template dilutions and the equation (1+e) = 10^(-1/slope)^, as described by Pfaffl [41]. Only primer pairs with an efficiency between 0.85 and 1.15, and a determination coefficient (R^2^) of the standard curve equal or superior to 0.985, were considered valuable. At the end of the amplification experiment, a melting curve was realized between 55°C to 95°C by steps of 0.5°C, to ensure that the signal corresponded to a single PCR product. For each target gene, PCR reactions were performed in triplicate from two first strand cDNA synthesis reactions; a biological repetition for the embryogenic genotype (mRNAs from 2 independent cultures), and a technical repetition for the non-embryogenic genotype (mixture of mRNAs from 3 cultures). The delta-delta cycle threshold (ΔΔC_T_) method for comparing the relative expression between genotypes was applied as described by Livak and Schmittgen [42], using as control a gene encoding actin-2 of chicory isolated in the laboratory (acc. num. DY800534). Data were subjected to analysis of variance for each gene (GLM procedure of SAS; [43]), using the following model Y_ij _= μ + G_i _+ S_j _+ e_ij_, with Y_ij _the ΔΔC_T_, μ the overall mean, G_i _the genotype effect, R_j _the repetition effect, and e_ij _the residual. The differential expression between the E and NE genotype was estimated using the least-square estimates of the means of the ΔΔC_T _computed from the model (LSMEANS option of the GLM procedure). Genes were considered as differentially expressed when the p-value associated with the F-value (Fisher-Snedecor) calculated for G_i _was <0.001, and when the mean ΔΔC_T _was ≤-0.6 or ≥0.6.

## List of abbreviations used

E/NE: embryogenic/non-embryogenic; EST: expressed sequence tag; SE: somatic embryogenesis; SSH: suppression subtractive hybridization

## Authors' contributions

SL prepared clones for sequencing, performed analyses and functional classification of ESTs, *in silico *subtraction and real-time RT-PCR analyses and drafted the manuscript. TH highly contributed to the interpretation of the results and to the redaction of the manuscript. JLH participated in planning and supervision of the study, and participated in the redaction of the manuscript. MCQ designed the study, collected plant tissues, performed RNA extractions and construction of the SSH libraries, participated in the redaction of the manuscript, and is the corresponding author. All authors read and approved the final manuscript.

## Note added in proof

During the process of submission, 38,323 EST sequences from *C. intybus *were added to the Compositae Genome Project Database, and the sequences of the 3,348 ESTs reported in this paper are now included in this database .

## Supplementary Material

Additional file 1 **Detailed data concerning ESTs assembling, annotation and classification of the 3348 ESTs**. In the first three columns, the accession numbers from GenBank of ESTs, their clone ID from our libraries, and their sequence length, are indicated. The columns 4 to 6 represent the assembling of identical ESTs into OCs (original clusters), with the OC ID, and the number of ESTs representing each OC in the E and the NE library. The field 'Contig' represents the assembling of EST corresponding to a same gene. 'Contig' includes the contig ID, and the number of ESTs representing each gene in the E and the NE library. The first hit from the BlastX search against the non-redundant Genbank database is provided for each OC. The detailed fields of the BlastX search against the NR Genbank database are ID, Putative function, Length, Score, E-value, Identity, Homology, Gap, Frame, and Alignment. The first hit from the BlastX search against the Arabidopsis translated coding sequences is provided for all ESTs. Detailed fields of this second BlastX search are ID, E-value, and the functional classification according to the MIPS functional catalogue. Click here for file

Additional file 2 **Perl scripts used for ESTs analyses**. Script 1: cleaning ESTs of vector and primers sequences. Script 2: local BlastN output parser. Script 3: BlastX output parser Click here for file

Additional file 3 **Primers used for real-time RT-PCR**. Oligonucleotide primers were designed for selected genes using the Beacon Designer software of Biosoft. Putative functions were defined according to BlastX searches against the non-redundant GeneBank database. Click here for file

## References

[B1] Zimmerman JL (1993). Somatic Embryogenesis: A Model for Early Development in Higher Plants. Plant Cell.

[B2] Feher A, Pasternak TP, Dudits D (2003). Transition of somatic plant cells to an embryogenic state. Plant Cell Tissue and Organ Culture.

[B3] Dubois T, Guerida M, Dubois J, Vasseur J (1991). Direct somatic embryogenesis in leaves of Cichorium. An histological and SEM study of early stages. Protoplasma.

[B4] Blervacq AS, Dubois T, Dubois J, Vasseur J (1995). First divisions of somatic embryogenic cells in Cichorium hybrid "474". Protoplasma.

[B5] Hendriks T, Scheer I, Quillet MC, Randoux B, Delbreil B, Vasseur J, Hilbert JL (1998). A nonsymbiotic hemoglobin gene is expressed during somatic embryogenesis in Cichorium. Biochim Biophys Acta.

[B6] Helleboid S, Chapman A, Hendriks T, Inze D, Vasseur J, Hilbert JL (2000). Cloning of beta-1,3-glucanases expressed during Cichorium somatic embryogenesis. Plant Mol Biol.

[B7] Galland R, Randoux B, Vasseur J, Hilbert JL (2001). A glutathione S-transferase cDNA identified by mRNA differential display is upregulated during somatic embryogenesis in Cichorium. Biochim Biophys Acta.

[B8] Randoux B, Quillet MC, Rambaud C, Vasseur J, Hilbert JL (2002). Identification of cDNAs encoding Rab-related GTP-binding proteins expressed during somatic embryogenesis in Cichorium. Plant Science.

[B9] Diatchenko L, Lau YF, Campbell AP, Chenchik A, Moqadam F, Huang B, Lukyanov S, Lukyanov K, Gurskaya N, Sverdlov ED, Siebert PD (1996). Suppression subtractive hybridization: a method for generating differentially regulated or tissue-specific cDNA probes and libraries. Proc Natl Acad Sci U S A.

[B10] Compositae Genome Project Database. http://cgpdb.ucdavis.edu/compositae.

[B11] Plant Genome Database. http://www.plantgdb.org/.

[B12] Bremer K (1994). Asteraceae cladistics & classification.

[B13] GeneBank database. http://www.ncbi.nlm.nih.gov/Genbank/index.html.

[B14] Arabidopsis thaliana Genome Database. http://www.plantgdb.org/AtGDB/.

[B15] Kirst M, Johnson AF, Baucom C, Ulrich E, Hubbard K, Staggs R, Paule C, Retzel E, Whetten R, Sederoff R (2003). Apparent homology of expressed genes from wood-forming tissues of loblolly pine (*Pinus taeda L.*) with Arabidopsis thaliana. Proc Natl Acad Sci U S A.

[B16] Barakat A, Szick-Miranda K, Chang IF, Guyot R, Blanc G, Cooke R, Delseny M, Bailey-Serres J (2001). The organization of cytoplasmic ribosomal protein genes in the Arabidopsis genome. Plant Physiol.

[B17] Bey M, Stuber K, Fellenberg K, Schwarz-Sommer Z, Sommer H, Saedler H, Zachgo S (2004). Characterization of antirrhinum petal development and identification of target genes of the class B MADS box gene DEFICIENS. Plant Cell.

[B18] Demura T, Tashiro G, Horiguchi G, Kishimoto N, Kubo M, Matsuoka N, Minami A, Nagata-Hiwatashi M, Nakamura K, Okamura Y, Sassa N, Suzuki S, Yazaki J, Kikuchi S, Fukuda H (2002). Visualization by comprehensive microarray analysis of gene expression programs during transdifferentiation of mesophyll cells into xylem cells. Proc Natl Acad Sci U S A.

[B19] (2000). The Arabidopsis Genome Initiative: Analysis of the genome sequence of the flowering plant Arabidopsis thaliana. Nature.

[B20] Lee NH, Weinstock KG, Kirkness EF, Earle-Hughes JA, Fuldner RA, Marmaros S, Glodek A, Gocayne JD, Adams MD, Kerlavage AR (1995). Comparative expressed-sequence-tag analysis of differential gene expression profiles in PC-12 cells before and after nerve growth factor treatment. Proc Natl Acad Sci U S A.

[B21] Okubo K, Hori N, Matoba R, Niiyama T, Fukushima A, Kojima Y, Matsubara K (1992). Large scale cDNA sequencing for analysis of quantitative and qualitative aspects of gene expression. Nat Genet.

[B22] Rajkovic A, Yan MSC, Klysik M, Matzuk M (2001). Discovery of germ cell-specific transcripts by expressed sequence tag database analysis. Fertil Steril.

[B23] Miner D, Rajkovic A (2003). Identification of expressed sequence tags preferentially expressed in human placentas by in silico subtraction. Prenat Diagn.

[B24] Bui LC, Leandri RD, Renard JP, Duranthon V (2005). SSH adequacy to preimplantation mammalian development: scarce specific transcripts cloning despite irregular normalisation. BMC Genomics.

[B25] Audic S, Claverie JM (1997). The significance of digital gene expression profiles. Genome Res.

[B26] Chapman A, Blervacq AS, Vasseur J, Hilbert JL (2000). Arabinogalactan-proteins in *Cichorium* somatic embryogenesis: effect of beta-glucosyl Yariv reagent and epitope localisation during embryo development. Planta.

[B27] Kidner CA, Martienssen RA (2005). The role of ARGONAUTE1 (AGO1) in meristem formation and identity. Dev Biol.

[B28] Dresselhaus T, Cordts S, Heuer S, Sauter M, Lorz H, Kranz E (1999). Novel ribosomal genes from maize are differentially expressed in the zygotic and somatic cell cycles. Mol Gen Genet.

[B29] Ranjan P, Kao YY, Jiang H, Joshi CP, Harding SA, Tsai CJ (2004). Suppression subtractive hybridization-mediated transcriptome analysis from multiple tissues of aspen (*Populus tremuloides*) altered in phenylpropanoid metabolism. Planta.

[B30] Matsubara S, Hurry V, Druart N, Benedict C, Janzik I, Chavarria-Krauser A, Walter A, Schurr U (2005). Nocturnal changes in leaf growth of Populus deltoides are controlled by cytoplasmic growth. Planta.

[B31] Feiler HS, Desprez T, Santoni V, Kronenberger J, Caboche M, Traas J (1995). The higher plant Arabidopsis thaliana encodes a functional CDC48 homologue which is highly expressed in dividing and expanding cells. Embo J.

[B32] McKhann HI, Frugier F, Petrovics G, de la Pena TC, Jurkevitch E, Brown S, Kondorosi E, Kondorosi A, Crespi M (1997). Cloning of a WD-repeat-containing gene from alfalfa (*Medicago sativa*): a role in hormone-mediated cell division?. Plant Mol Biol.

[B33] Lynn K, Fernandez A, Aida M, Sedbrook J, Tasaka M, Masson P, Barton MK (1999). The PINHEAD/ZWILLE gene acts pleiotropically in Arabidopsis development and has overlapping functions with the ARGONAUTE1 gene. Development.

[B34] Baldwin TC, Domingo C, Schindler T, Seetharaman G, Stacey N, Roberts K (2001). DcAGP1, a secreted arabinogalactan protein, is related to a family of basic proline-rich proteins. Plant Mol Biol.

[B35] Showalter AM (2001). Arabinogalactan-proteins: structure, expression and function. Cell Mol Life Sci.

[B36] Buchanan-Wollaston V, Earl S, Harrison E, Mathas E, Navabpour S, Page T, Pink D (2003). The molecular analysis of leaf senescence - a genomics approach. Plant Biotechnology Journal.

[B37] Kunieda T, Fujiwara T, Amano T, Shioi Y (2005). Molecular cloning and characterization of a senescence-induced tau-class Glutathione S-transferase from barley leaves. Plant Cell Physiol.

[B38] BioEdit sequence alignment editor. http://www.mbio.ncsu.edu/BioEdit/bioedit.html.

[B39] Munich Information Centre for Proteins Sequences, functional catalogue. http://mips.gsf.de/projects/funcat.

[B40] Significance of digital gene expression profiles. http://www.igs.cnrs-mrs.fr/Winflat/winflat.cgi.

[B41] Pfaffl MW (2001). A new mathematical model for relative quantification in real-time RT-PCR. Nucleic Acids Res.

[B42] Livak KJ, Schmittgen TD (2001). Analysis of relative gene expression data using real-time quantitative PCR and the 2(-Delta Delta C(T)) Method. Methods.

[B43] SAS intitute Inc.99. SAS/STAT guide for personal computers, v8.2 ed.

